# Factors Influencing Adult Physical Health after Controlling for Current Health Conditions: Evidence from a British Cohort

**DOI:** 10.1371/journal.pone.0066204

**Published:** 2013-06-24

**Authors:** Helen Cheng, Adrian Furnham

**Affiliations:** Department of Psychology, University College London, London, United Kingdom; University of São Paulo, Brazil

## Abstract

This study explored a longitudinal data set of 6875 British adults examining the effects of parental social status (measured at birth), cognitive ability (at age 11 yrs), personality traits, education and occupational attainment on physical health and functioning (all measured at age 50 yrs), after taking account of current health conditions (number of illness). Correlation analysis showed that parental social class, childhood cognitive ability, education and occupation, and two personality traits (Emotional Stability/Neuroticism, and Conscientiousness) were all significantly associated with adult physical health variables. Structural equation modelling showed that health conditions and personality traits were significantly, and inversely, associated with physical health (indicated by good daily physical functioning, relative absence of pain, perceived health, and low level of limitations at work due to physical health). Parental social status, childhood intelligence, educational and occupational attainment were all modestly, but significantly and directly, associated with adult physical health. The effect of childhood intelligence on adult physical health was, in part, mediated through Emotional Stability and Conscientiousness. After controlling for health conditions Emotional Stability was the strongest predictor of physical health. Implications and limitations are discussed.

## Introduction

Physical health and functioning affects all aspects of human activities such as work obligations, family roles, social activities and engagement, as well as psychological health and well-being. The determinants of health are manifold: socioeconomic, educational, genetic, psychological, and environmental. This study examined the effects of parental socioeconomic status, childhood intelligence, two personality traits, education, occupation and current health on four facets of physical health.

There are established links between income inequality and health [1]–[3] and social status and health [4], [5]. In general, people from lower socioeconomic status groups have worse physical (and mental) health than those above them 6. Mental health and physical health are correlated and chronic diseases and mortality rates are higher among patients with mental health disorders than in the general population [7]–[9].

Whilst many studies examined the associations between personality traits and mental health and well-being [10]–[14] fewer studies have looked at the associations between personality traits and physical health. However studies that have examined health status and the Big Five personality traits have always identified two; namely Conscientiousness and Neuroticism as consistently and directly related to numerous different medical disorders [15], [16] and between Conscientiousness and reduced mortality [17]. For this reason, these two specific traits will be examined in this study.

In recent years a number of studies have shown the associations between trait Conscientiousness and physical health [18]–[21]. In a study, researchers conducted a meta-analysis of the association between Conscientiousness-related traits and longevity [22]. Using a random-effects analysis model on 20 independent samples, they found that higher levels of Conscientiousness were significantly and positively related to longevity (*r = *.11), suggesting the importance of Conscientiousness-related traits to health across the life span. Others found, as predicted, that trait Conscientiousness significantly predicted greater longevity, even in a model where gender, age and years of education were controlled [23]. There is also evidence that conscientiousness mediates the relationship between parental socialisation and self-reported health [24].

Studies on the Big Five personality traits have examined ethnicity, gender and racial differences. They have typically show small, but predictable, differences between the sexes (females scoring higher on Agreeableness and Neurotocism) and cultures [25]. There have also been studies on the measurement of the Big Five in children [26] as well as the stability of personality over time [27]. They suggest it is possible to validly assess the Big Five traits in young children, and that personality seems most stable between the ages of 30 and 60 yrs particularly using established big five measures to assess it. There are modest increases in Emotional Stability and Agreeableness over this period with Extraversion and Neuroticism showing least change (both with a slight decline) and Conscientiousness showing most change (an increase). Males seem more stable than females. This literature therefore suggests that studies such as this that examine Big Five personality correlates of physical health are generalisable across different populations groups.

Various studies have shown the links between family socio-economic status in childhood and educational achievement in adulthood [28]–[30]. Family socioeconomic status in childhood is associated with children’s early cognitive development [31], and childhood intelligence is linked with later educational and occupational attainment [32]–[35]. Further, it has been shown that parents’ mental health and parent-child relationships have a direct effect on their children’s cognitive and social development as well as their mental health in later life [36], [37]. Also, intelligence has also been found to be associated with physical health and reduced mortality [38]–[40].

Two personality traits, Emotional Stability (low Neuroticism, high adjustment) and Conscientiousness (prudence), and intelligence have been found to be consistently associated with social stability and career success [41]. Socio-economic status, education, occupation, personality and measures of physical health have been found to be inter-correlated in many previous studies. It is therefore important to examine these factors *together* to determine to what extent each factor is independently associated with physical health.

This study sets out to explore the associations between personality traits and physical health, taking into account the effects of parental social status, childhood intelligence, educational and occupational attainment, and current health conditions, using a path model and drawing on data collected from a large representative population sample born in 1958. One unexplored question is how two well-established personality factors can increase the explained variance over and above intelligence, demographic and occupational factors in explaining physical health.

Based on previous findings it is hypothesised that a) childhood intelligence would be associated with emotional stability and conscientiousness; b) childhood intelligence would be associated with health conditions and physical health; c) parental social status, education, and own current occupation would be associated with adult health conditions and current physical health; and, d) emotional stability and conscientiousness would be positively associated with physical health.

First we look at the associations between the measures used in the study. Following this we will test two models: Model 1 examined the effects of parental social status, childhood intelligence, educational qualifications and current occupational levels, and health conditions on adult physical health; and model 2 investigated the paths linking all measures used in model 1 together with personality factors using structural equation modelling.

## Methods

### Participants

The National Child Development Study (NCDS) 1958 is a large-scale longitudinal study of all 17,415 individuals who were born in Great Britain in a week in March 1958 [42]. In the NCDS participants were recruited as part of a perinatal mortality survey (The dataset is available in http://ukdataservice.ac.uk/). As far as the authors are aware ethical practices were engaged as per NCDIS policies and procedures. The following analysis is based on data collected when the study participants were tested for their general cognitive abilities at age 11, and at age 50 years. Participants responded to a set of questionnaires including personality traits and a number of physical health measures, with information on educational qualifications they obtained and current occupational prestige. 14,134 children at age 11 completed tests of cognitive ability (response = 87%). Testing took place in school, and written, informed consent was given by the parents. At 50 years, 9,760 participants answered questions about their health in general, and whether they currently suffered from a number of problems listed on a card (response = 79%), and 8,508 participants completed a questionnaire on personality traits and a number of physical health measures (response = 69%). The analytic sample comprises 6,875 cohort members (51 per cent females) for whom complete data were collected at birth, at age 11, and the outcome measures at age 50. Analysis of response bias in the cohort data showed that the achieved adult samples did not differ from their target sample across a number of critical variables (social class, parental education and gender), despite a slight under-representation of the most disadvantaged groups [43]. Bias due to attrition of the sample during childhood has been shown to be minimal [44]–[46].

### Measures

#### Family social status at birth

Family social status was assessed through parental occupational social status and parental education. Parental occupational status at birth was measured by the Registrar General’s measure of social class (RGSC), defined according to occupational status and the associated education, prestige and lifestyle [47]. Where the father was absent, the social class RGSC of the mother was used. RGSC was coded on a six-point scale: I professional; II managerial\tech; IIIN skilled non-manual; IIIM skilled manual; IV semi-skilled; and V unskilled occupations [48]. Parental education is measured by the age either parent had left full-time education.

#### Childhood cognitive ability

Cognitive ability was assessed at age 11 in school using a general ability test [49] consisting of 40 verbal and 40 non-verbal items. Children were tested individually by teachers, who recorded the answers for the tests. For the verbal items, children were presented with an example set of four words that were linked either logically, semantically, or phonologically. For the non-verbal tasks, shapes or symbols were used. The children were then given another set of three words or shapes or symbols with a blank. Participants were required to select the missing item from a list of five alternatives. Scores from these two set of tests correlate strongly with scores on an IQ-type test used for secondary school selection suggesting a high degree of validity 32. The predictive validity of the childhood cognitive ability tests is reported using the same dataset (e.g. r = .48, p<.00) between the combined scores of childhood cognitive ability and educational achievement in adulthood, (Schoon, 2010; r = .30 and r = .29 p<.001 )between childhood verbal and non-verbal tests and adult earning respectively for men and r = .26 and r = .23 p<.001 for women [50]. There is also evidence that cognitive ability is very stable over time 32.

#### Educational qualifications and occupational attainment

At age 50, participants were asked about their highest academic or vocational qualifications. Responses were coded to the six-point scale of National Vocational Qualifications levels (NVQ) which ranges from ‘none’ to ‘higher degree level’ Data on current or last occupation held by cohort members at age 50 were coded according to the RGSC described above, using a 6-point classification.

#### Personality traits

Personality traits were assessed by the 50 questions from the International Personality Item Pool (IPIP) [51]. Responses (5-point, from “Strongly Agree” to “Strongly Disagree”) are summed to provide scores on the so called ‘Big-5′ personality traits: Extraversion, Emotionality, Conscientiousness, Agreeableness and Intellect (Conscientiousness). Scores on each trait range between 5 and 50 with higher scores equating to higher levels of each trait. Based on the literature in the area, among the five personality traits, Emotionality and Conscientiousness appeared to be significantly associated with the outcome variables. A preliminary analysis on the dataset confirmed this, therefore these two personality traits were used in the study. The internal consistency (alpha coefficient) was 0.88 for Emotionality, and 0.77 for Conscientiousness.

#### Health conditions and physical health measures

Health conditions were assessed by questions used in the follow-up interview when cohort members were 50 years old Participants were asked whether they currently suffer from a number of health conditions listed on a card (interviewers were instructed to exclude temporary conditions). A variable of health conditions was created using fifteen health conditions listed on the card (asthma, hay fever, diabetes, high blood pressure, migraine, chronic fatigue, cancer or leukaemia etc.) ranging from 0 to 9 (0 = no such conditions to 9 types of physical conditions listed on the card). Physical health measures include four scales used in the cohort study: scale of daily physical functioning, scale of limitations at work due to physical health, pain measure, and perceived health measure. Scale of daily physical functioning comprises of 10 items and participants were asked whether their health limited them in these activities (3-point, from “Limited a Lot” to “Not Limited at All”). Item examples are “lifting or carrying groceries”, “climbing several flights of stairs”, “bathing or dressing yourself”; Scale of limitations at work due to physical health comprises 4 items (Yes/No). Item examples are “Health led to less time on work/activities in past 4 weeks”, and “Physical health led to accomplish less than liked in last 4 weeks”; Pain was assessed by two questions “How much bodily pain had during the past 4 weeks?” (5-point, from “None” to “Severe or Very Severe”) and “How much did pain interfere with normal work in past 4 weeks?” (5-point, from “not at all” to “extremely”). Scores of the two items were combined for analysis; Scale of perceived health comprises of 4 items (5-point, from “Definitely True” to “Definitely False”). Item examples are “I seem to get ill a little easier than other people”, and “My health is excellent”; and The internal consistency (alpha) was 0.94 for daily physical functioning, 0.91 for limitations at work due to physical health, 0.86 for pain measure, and 0.79 for perceived health.

### Statistical Analyses

First we conducted a Pearson correlational analysis between the measures used in the study. SPSS version 18 is used for this analysis. Following this we tested two models described above using structural equation modelling. AMOS version 18 is employed for the model testing.

## Results

### Correlational Analysis

Table 1 shows the correlations between the observed variables in the study, together with the means and standard deviations of the measures. Higher scores on parental social status and childhood cognitive ability indicators, higher levels of educational qualifications and occupational prestige, and higher scores on Emotional Stability and Conscientiousness were all significantly and positively associated with daily physical functioning and perceived health and negatively associated with limitations at work due to physical health and pain. Childhood cognitive ability tests were significantly and positively associated with parental social status indicators, educational qualifications occupational prestige, and personality traits.

**Table 1:** Pearson correlations childhood cognitive ability, personality traits, physical health variables, and demographic variables.

	*Variables*	Mean (SD)	1	2	3	4	5	6	7	8	9	10	11	12	13	14	15
1.	Gender	.51 (.50)	_														
2.	Parentalsocial class	3.27 (1.23)	−.010	_													
3.	Paternaleducation	15.48 (1.95)	.020	.470	_												
4.	Maternaleducation	15.49 (1.56)	.036	.356	.518	_											
5.	Verbal scores(cognitive ability)	23.87 (8.82)	.115	.274	.247	.223	_										
6.	Non-verbal scores(cognitive ability)	22.37 (7.13)	.012	.278	.243	.209	.788	_									
7.	Educationalqualification	2.65 (1.38)	−.016	.254	.249	.225	.437	.415	_								
8.	Occupationalprestige	4.08 (1.22)	.001	.207	.181	.156	.326	.309	.427	_							
9.	Healthconditions	1.70 (1.30)	.043	−.042	−.049	−.044	−.048	−.040	−.078	−.030	_						
10.	Physicalfunctioning	27.31 (4.25)	−**.079**	**.102**	**.110**	**.084**	**.160**	**.160**	**.175**	**.096**	−**.300**	_					
11.	Limitations atwork due tophysical health	.68 (1.32)	**.056**	−**.045**	−**.069**	−**.041**	−**.053**	−**.071**	−**.069**	−**.049**	**.277**	−.543	_				
12.	Pain	2.09 (2.14)	**.068**	−**.091**	−**.090**	−**.067**	−**.096**	−**.106**	−**.132**	−**.097**	**.356**	−.589	665	_			
13.	Perceivedhealth	15.22 (3.50)	**.030**	**.081**	**.079**	**.068**	**.086**	**.088**	**.100**	**.059**	−**.393**	.546	−.526	−.565	_		
14.	Emotionality	28.46 (7.25)	−.127	.037	.038	.019	.085	.121	.105	.079	−.193	**.200**	**.232**	−**.267**	**.335**	_	
15.	Conscientiousness	33.78 (5.44)	.097	.053	.037	.051	.081	.069	.100	.099	−.094	**.154**	**.125**	−**.136**	**.239**	.218	_

Note: Variables were scored such that a higher score indicated being female, a more professional occupation for the parent and higher age parents left school, a higher verbal and non-verbal ability scores, highest educational qualification, more professional occupation, a higher scores on health conditions, a higher score on physical health variables, a higher score on Emotional Stability, and a higher score on Conscientiousness. The numbers in bold are the correlation coefficients between the outcome measures and other covariates.

### Structural Equation Modelling

Structural Equation Modelling (SEM) was used to assess the links among family social background, childhood cognitive ability indicators, adult educational qualifications and occupational prestige, personality traits, health conditions, and physical health and functioning measures. Paths in the models are designed to correspond with the time sequence in which the variables occurred, as well as following the rationale that more “stable” variables predict more “changeable” variables. The SEM model testing was carried out using the structural equation modelling program AMOS 18 [52]. The AMOS program uses maximum likelihood estimation that can be based on incomplete data, known as the full information maximum likelihood (FIML) approach. FIML is preferable to maximum likelihood estimation based on complete data (the listwise deletion (LD) approach) since FIML estimates tend to show less bias and are more reliable than LD estimates even when the data deviate from missing at random and are non-ignorable [53].

**Figure 1:**
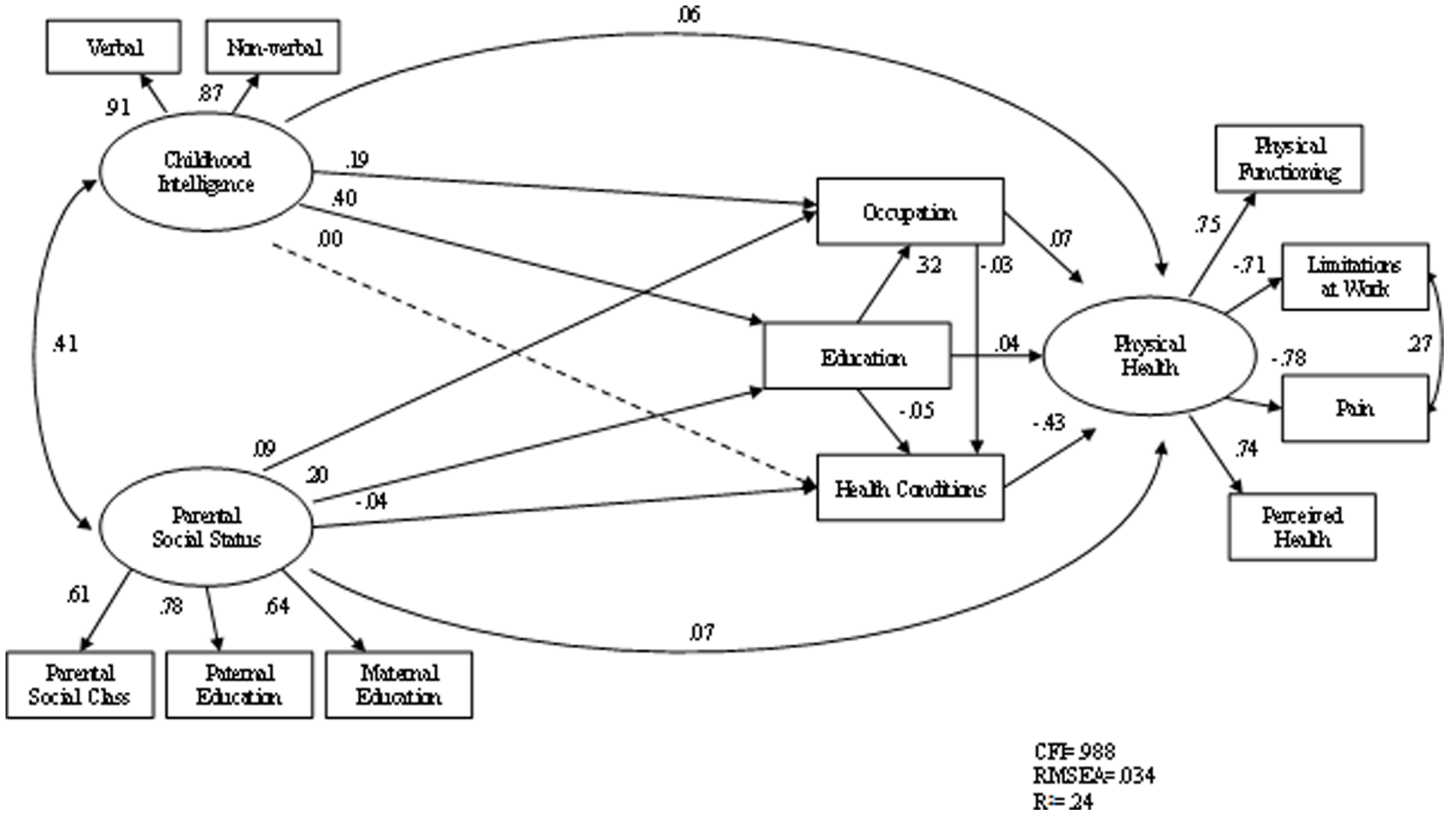
Path model of physical functioning without personality factors (N = 6875).

**Figure 2:**
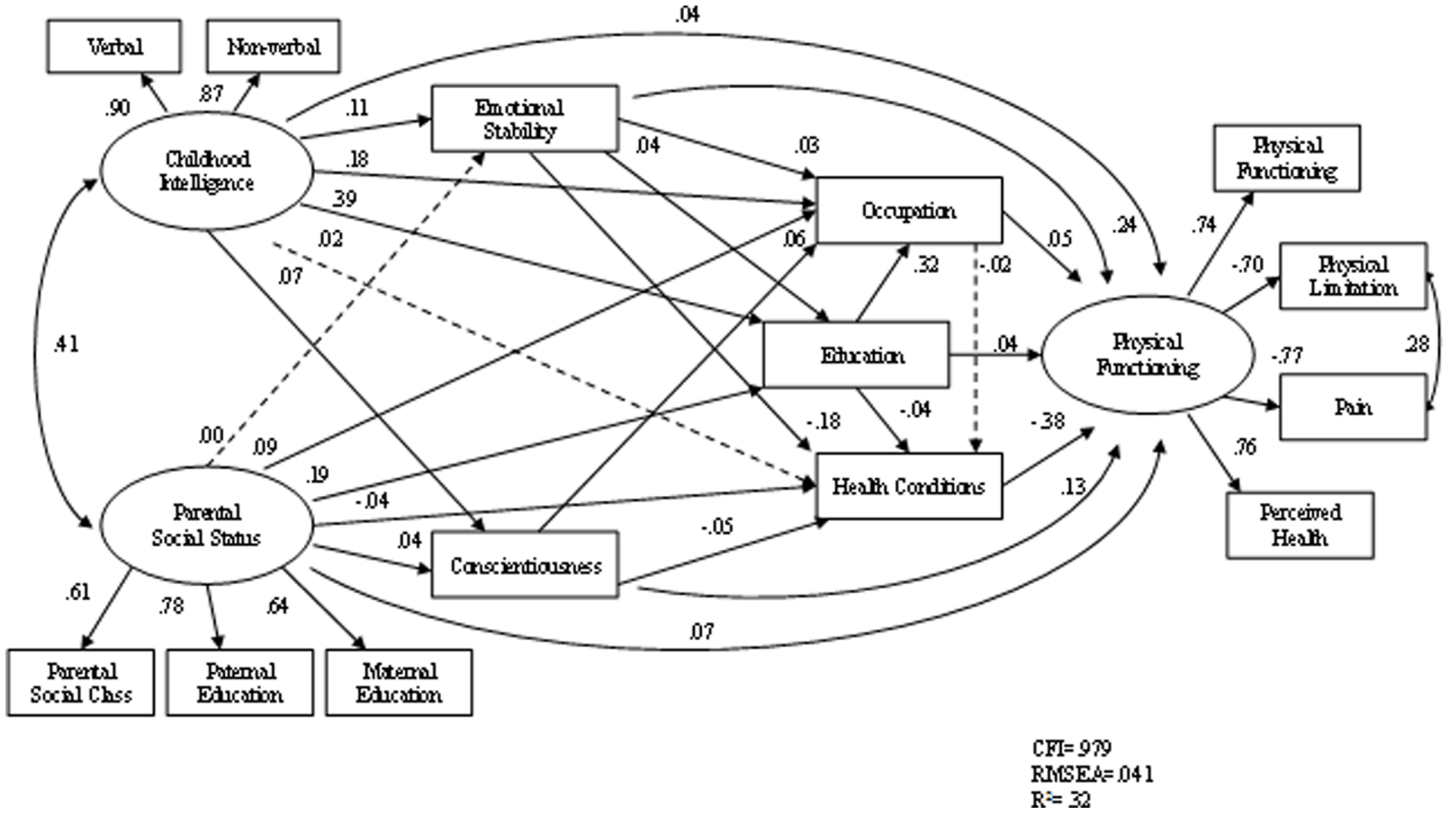
Path model of physical functioning with personality factors (N = 6875).

Figure 1 and Figure 2 show the standardised path coefficients of the structural equation models. The usual structural equation modelling conventions are used, with the latent variable shown as a circle and manifest variables in rectangles. Single headed arrows represent causal influences. The double-headed arrow represents the correlation between independent variables. The solid lines indicate the corresponding path coefficients are statistically significant and dashed indicate the path coefficients are non-significant. Unique and error variance for each observable variables are included in the model (omitted in the models). From the modification indices, the residuals between limitations at work due to physical health and pain were allowed to co-vary (correlated errors) to improve model fit. Path estimates are given as standardised regression coefficients. Gender was controlled in both models in Figures 1 and 2 (not shown in the diagrams).

#### Model fit

The χ^2^ statistic is overly sensitive to model misspecification when sample sizes are large or the observed variables are non-normally distributed. The root mean square error of approximation (RMSEA) gives a measure of the discrepancy in fit per degrees of freedom (<.05 indicates a good fit). The comparative fit index (CFI) where values above .95 indicate good fit [54].

Model 1 showed a good fit. Chi-square was 401.3 (*df* = 41, *p*<.001), the CFI was .986, and the RMSEA was .036. The model explains 24 per cent of the total variance, 95% CI [.21, .27]. Figure 1 shows parental social status, childhood intelligence, educational qualifications and current occupational attainment all were directly and significant associated with adult physical health and functioning, though these associations were modest. The effects of parental social status and childhood intelligence on adult physical health were in part mediated through educational qualifications and own occupational levels. Understandably, the strongest association in the model was between current health conditions and physical health.

Model 2 also showed a good fit. Chi-square was 686.3 (*df* = 53, *p*<.001), the CFI was .977, and the RMSEA was .042. The model explains 32 per cent of the total variance (8 per cent increase compared with model 1 shown in Figure 1) in adult physical health, 95% CI [.29, .35]. The f^2^ values of .02, .15, and .35 are termed small, medium, and large, respectively [55]. Figure 2 shows that after entering the two personality traits into the model, all predictors in model 1 shown in Figure remained modestly but significantly associated with the outcome variable. Personality traits Emotional Stability and Conscientiousness were directly associated with physical health outcome, and the effect of emotional stability on physical health was in part mediated through health conditions. The effect of childhood intelligence on adult physical health was in part mediated through emotional stability. Apart from health conditions, the strongest path coefficient was between emotional stability and physical health.

## Discussion

This study is among the first longitudinal, population based research, to explore the associations between personality traits and physical health, specifically taking into account the effects of parental social status, childhood intelligence, educational and occupational attainment, and current health conditions. It shows that personality traits, intelligence, and social factors are all significant factors that influence physical health and functioning. Whilst the study confirms the previous findings of the associations between intelligence and physical health, it provides evidence of the independent associations between personality traits and physical health. It provides yet more evidence of the necessity of taking a biopsychosocial approach to medical problems [56]. What is new about this study can be seen in the difference between figures 1 and 2 where we show that two personality traits can account for an additional 18% in the explained variance.

All four hypotheses were confirmed. Parental social status, educational qualifications and occupational levels were all independent predictors of adult physical health. Participants who were from lower socioeconomic background, who had lower educational qualifications and were in lower occupational positions were more likely to suffer from health conditions, and tended to have worse physical health than those who came from a more privileged family background, had higher educational qualifications and in a higher social position as found in previous studies. Furthermore, childhood cognitive ability, which was significantly associated with parental social-economic conditions, did not only influence people’s educational achievement and occupational prestige later on, but also could affect their traits Emotional Stability and Conscientiousness and thus consequently physical health in their adulthood.

Conscientiousness is associated with prudence, reliability and rule following. People who scored higher on this trait tend to be more cautious than risk-taking in their daily life with all aspects of their health. Researchers reviewed 194 studies and found that Conscientiousness related traits were negatively related to all risky health-related, and positively related to all beneficial health-related behaviours [57]. In this study Conscientiousness was a significant and direct correlate of physical health functioning. It is, no doubt, the facets of self-discipline and deliberation that best explain the positive association between Conscientiousness and both mental and physical health.

Emotional Stability (or high adjustment, low Neuroticism) was the second strongest predictor of adult physical health in the model following current health conditions. People with Neuroticism are prone to anxiety, depression, hypochondriasis and pessimism and are more likely to both report and suffer from health conditions. There is considerable evidence that Neuroticism is a major risk factor for multiple diseases. Some suggest that this is due to Neuroticism being associated with loss of immune system functioning and higher stress responses [16]. They note it is associated with a “wide range of poorer healthy outcomes” (p322). Neuroticism is also associated with a lower incidence of effective stress coping strategies as well as an unhealthy lifestyle (smoking and drinking). What is particularly interesting in this study is evidence of the direct power of trait Neuroticism on physical functioning, though this may in part be due to ‘complaint-proneness’ and ‘distress over-reporting’ associated with Neuroticism.

As with all research using cohort studies, this work is constrained by of the availability of the data, thus restricting the potential mechanisms and processes, which we can examine. Another limitation is the attrition of respondents over time. Response bias at the individual level would tend to underestimate the magnitude of the effects of social family background on future development since sample attrition is greatest amongst individuals in more deprived circumstances. Our results may thus be a conservative estimate of the long-term influence of social inequalities experienced during childhood. Missing data at the variable level may also be non-random. The FIML approach has been adopted for dealing with these problems, but bias in our model estimates may still be present. Further, there is always the problem of potential biases in self-report data. That is, participants may be reluctant to disclose their medical illnesses to researchers especially if they are unconfirmed by secondary sources or medical diagnoses.

Ideally we would have liked personality traits to have been measured earlier though there is considerable evidence of the stability of personality traits over adulthood [58]. There is a considerable debate about change and stability in personality over time with evidence that Neuroticism is fairly stable over adulthood (30–70 years), though Conscientiousness does increase [59]. It is of course possible that very poor physical health over time may effect personality, though the literature seems to suggest the direction of causality is primarily from traits to health and not vice versa [18]. Again, we are restricted by the availability of the data to test the stability of personality traits in the study. Although more than a third of the total variance of physical health is accounted for, nearly two-thirds of the variance remains unexplained. Future research may explore these factors together with environmental factors that affect physical health. Further studies are required to examine the mechanism and processes of socioeconomic, psychological, and environmental factors, so that the causal directions and interactions among these factors can be better understood, and to effectively reduce the inequality in health in the society.

Certainly if better understood these findings regarding personality and physical health could have implications for targeted surveillance of certain types, more effective intervention strategies as well as improvements in doctor-patient communication.
